# Plasma Shielding Effect in Nanosecond/CW Combined Pulse Laser Ablation of Metals

**DOI:** 10.3390/ma19061117

**Published:** 2026-03-13

**Authors:** Xianshi Jia, Yuehao Cai, Junyang Xu, Lu Zhang, Kai Li, Xin Li, Ke Sun, Zhou Li, Cong Wang

**Affiliations:** 1 State Key Laboratory of Precision Manufacturing for Extreme Service Performance, College of Mechanical and Electrical Engineering, Central South University, Changsha 410083, China; 221026@csu.edu.cn (X.J.);; 2 State Key Laboratory of Pulsed Power Laser Technology, College of Electronic Engineering, National University of Defense Technology, Hefei 230037, China; 3 Anhui Laboratory of Advanced Laser Technology, Hefei 230037, China; 4 Nanhu Laser Laboratory, Changsha 410073, China; 5 School of Intelligent Manufacturing, Hunan First Normal University, Changsha 410221, China

**Keywords:** metal ablation, combined pulse laser, plasma shielding effect, laser–material interaction

## Abstract

Combined pulse laser systems combining continuous-wave (CW) lasers and nanosecond pulsed lasers have shown clear advantages in metal ablation and surface modification. However, the plasma shielding effect induced by nanosecond pulses and the associated shock-wave phenomena in hybrid laser systems remain insufficiently investigated, particularly regarding their influence on CW laser energy coupling. In this study, the ablation behavior of metal targets under the combined irradiation of a 500 W CW laser and nanosecond pulsed lasers with pulse energies ranging from 0.4 J to 1.0 J was investigated. High-speed plasma imaging was employed to analyze laser–material interaction characteristics, including absorption behavior and molten material ejection, while high-speed infrared thermography was used to monitor transient temperature evolution during combined pulse laser processing. Macroscopic and microscopic analyses were conducted to characterize damage morphology, and a three-dimensional surface profilometer was used to quantitatively evaluate ablation efficiency. The results show that, under combined pulse laser irradiation, the removed volume increased from 0.05 mm^3^ to 0.618 mm^3^ and the ablation depth increased from 0.136 mm to 0.776 mm. Compared with CW laser processing alone, the ablation efficiency was markedly enhanced. This improvement is attributed to the combined effects of optimized energy deposition, thermal distribution, and material response. In addition, the plasma shielding effect was observed to vary with nanosecond pulse energy, indicating that precise energy control is critical for performance enhancement. This study demonstrates the potential of combined pulse laser technology for high-efficiency and high-precision metal surface processing and micro–nano fabrication.

## 1. Introduction

Laser processing is a mature and versatile manufacturing technology that is gradually replacing certain conventional processing methods due to its wide application range and cost-effectiveness [1,2,3]. With the increasing adoption of laser processing in consumer electronics, automotive manufacturing, aerospace, and medical applications, the global laser market has experienced increasingly diversified demands [4,5,6,7,8]. Among various laser processing technologies, laser ablation has long been regarded as a fundamental research topic and has been extensively and systematically investigated in laser interactions with metals [9], semiconductors, transparent materials [10], and optical coatings [11]. Improving laser ablation performance remains an important objective in this field, and combined pulse laser ablation techniques have demonstrated considerable potential in this regard [12,13,14]. Previous studies have shown that, in the ablation of transparent materials, nanosecond laser pre-irradiation can introduce defects or molten regions, while ultrafast lasers can enhance transient activation zones, thereby improve material absorption of subsequent laser irradiation and optimizing ablation efficiency [15,16,17,18]. In addition, increasing the removal efficiency of molten material and suppressing thermal effects during drilling processes are also effective approaches for enhancing ablation performance [19]. In metal ablation, recoil pressure and strong shock waves induced by auxiliary nanosecond or ultrafast lasers have been reported to facilitate molten material expulsion and strengthen ablation performance. With the successful improvement of ablation efficiency achieved in metal target processing using combined pulse laser techniques, this approach has attracted increasing research interest [20].

Combined pulse laser ablation techniques typically integrate two or more laser pulses with different pulse durations, wavelengths, or energies, and achieve synergistic enhancement of ablation efficiency and processing quality through precise temporal and spatial control [21]. The underlying mechanism relies on the use of a leading pulse to precondition the material surface, such as through preheating, oxidation, defect introduction, or plasma generation, thereby modifying the optical and thermal properties of the material and enhancing energy absorption and ablation response to the subsequent main pulse [22]. For example, nanosecond laser irradiation can induce microstructures or molten layers on metal surfaces, which improves the absorption efficiency of subsequent ultrafast laser pulses [23,24,25,26,27]. In contrast, femtosecond or picosecond lasers enable so-called “cold processing” owing to their extremely short pulse durations, significantly reducing the heat-affected zone and improving machining precision [28,29,30]. By combining long- and short-pulse lasers, combined pulse laser systems can simultaneously benefit from the high energy deposition efficiency of long pulses and the high precision with low thermal damage characteristic of ultrafast pulses, thereby achieving a balance between high processing efficiency and fine structural control.

In the field of combined pulse laser-induced damage, including CW–nanosecond pulse combinations, nanosecond–picosecond short-pulse sequence combinations, and nanosecond laser coupling with other energy fields such as electric or magnetic fields [31], most studies have primarily focused on the evolution of macroscopic damage characteristics, such as damage threshold, damaged area, and damage depth, as functions of nanosecond pulse energy. However, the dynamic relationship between plasma generation induced by nanosecond laser irradiation and the resulting damage mechanisms of the target material has not been sufficiently explored [32]. In particular, limited attention has been paid to the differences in plasma effects induced by nanosecond laser pulses across different energy regimes, as well as to the mechanisms by which plasma shielding induced at different pulse energies influences damage efficiency [33,34,35,36,37,38]. In the field of combined pulse laser drilling, nanosecond lasers are commonly employed as pre-ablation sources, auxiliary drilling sources, or primary drilling sources; nevertheless, their intrinsic effects on drilling quality and efficiency remain unclear [39,40,41,42,43]. Some studies have applied nanosecond laser pre-ablation to target surfaces and reported a reduction in the drilling threshold for subsequent laser irradiation [44]. However, the shielding effect of plasma generated during nanosecond laser pre-ablation on subsequent laser energy absorption has not been systematically analyzed, nor has the relationship between nanosecond pulse energy, pre-ablation layer thickness, and plasma density been quantitatively established. As a result, the design of pre-ablation parameters lacks a solid theoretical basis, limiting precise process control [45,46,47]. Other studies have combined nanosecond lasers with ultrafast lasers for high-aspect-ratio drilling and mainly emphasized the efficiency advantages over single-laser drilling techniques [48,49,50], while overlooking the interaction behavior between nanosecond laser-induced plasma and ultrafast laser-induced plasma, as well as the evolution of plasma shielding effects arising from such interactions. This limitation has constrained further improvements in drilling quality using combined pulse laser techniques [51].

In the representative combined pulse laser damage scenario involving the combined irradiation of CW and nanosecond pulsed lasers for metal ablation, most existing studies have primarily focused on the evolution of macroscopic ablation efficiency, such as ablation rate and removed volume. However, the dynamic evolution characteristics of plasma induced by nanosecond pulses at different energy levels [52] have rarely been correlated with the transient thermal response of the target material, including heating rate, peak temperature, and cooling rate, as well as with microscopic ablation morphology features such as crater dimensions and edge protrusions. The lack of such multidimensional correlation analysis has hindered a clear understanding of the specific mechanisms by which plasma shielding effects influence ablation behavior and damage efficiency in combined pulse laser systems [53].

Therefore, in this study, an experimental system for metal target ablation under the combined irradiation of a 500 W CW laser and a nanosecond pulsed laser was established, with 304 stainless steels selected as the target material. High-speed infrared thermography, high-speed imaging, and three-dimensional surface morphology characterization techniques were integrated to systematically investigate the effects of nanosecond pulse energy (0.4–1.0 J) on plasma evolution, transient temperature response, and ablation performance. The objective of this work is to clarify the ablation mechanisms of metal targets in combined pulse laser systems and to elucidate the role of plasma shielding effects induced by nanosecond pulses in governing ablation efficiency.

## 2. Materials and Methods

**Figure 1:**
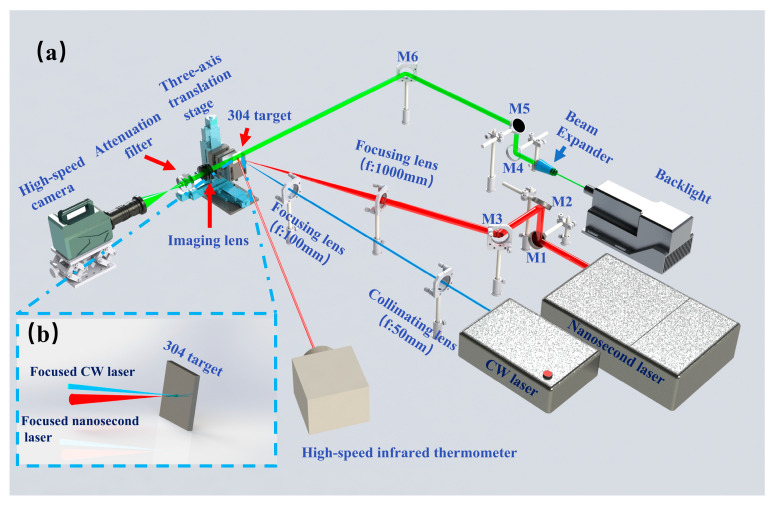
Experimental setup and laser configuration: (**a**) Schematic illustration of the experimental setup; (**b**) Schematic diagram of combined pulse laser focusing on the metal target.

This section details the research methodology, as shown in Figure 1. Figure 1a shows the schematic diagram of the experimental setup used in this study, while Figure 1b illustrates the focusing configuration of the combined pulse laser on the target surface. The experimental system consisted of a CW fiber laser with a wavelength of 976 nm and a core diameter of 600 μm (BWT, Beijing, China), and a nanosecond pulsed laser with a pulse duration of 10 ns, a wavelength of 1064 nm, and a repetition rate of 5 Hz (BEAMTECH, Beijing, China). As shown in Figure 1a, the CW laser beam was first collimated using a collimating lens and subsequently focused onto the target surface through a focusing lens with a focal length of 100 mm ensuring the CW laser focus was perfectly coincident with the sample’s upper surface and forming a stable irradiation spot at this position. Meanwhile, the nanosecond pulsed laser beam with an angle of 15° relative to the CW laser beam was reflected by mirrors M1–M3 and then focused onto the target surface using a focusing lens with a focal length of 1000 mm. To achieve spatial coincidence between the two laser beams, the nanosecond laser was first operated at a low pulse energy for focus adjustment: the optical path of the nanosecond laser was fine-tuned by adjusting the reflective mirrors M1–M3 and the three-axis translation stage, until the action center of the nanosecond laser was completely aligned with that of the CW laser on the sample surface. After the spatial coincidence of the two laser beams was confirmed, high-energy nanosecond laser pulses were used for the subsequent combined laser ablation experiments. The nanosecond laser induced a plasma filament with a length of ~30 mm under the experimental conditions, and the middle part of the filament along its axial direction was precisely coincident with the upper surface of the sample (the molten surface formed by CW laser irradiation). During CW laser heating, the molten pool depth (0.1–0.5 mm) remained much smaller than the filament length; therefore, the molten surface stayed within the effective focal region of the ns beam. Consequently, the nanosecond pulses interacted near the focal plane of the CW-induced molten surface.

Combined pulse laser ablation of metals is a complex physical process involving multiple coupled phenomena. To gain a comprehensive understanding of the ablation dynamics and the damage mechanisms of the metal target, an integrated diagnostic system was employed in this study. The post-ablation surface morphology was characterized using a three-dimensional surface profiler (KEYENCE VR-6100, Osaka, Japan), which enables surface, cross-sectional, and three-dimensional measurements. Key ablation features, including surface morphology, ablation diameter, depth, and volume, were quantitatively analyzed. A high-speed imaging system (SSZN SH3 103, Shenzhen Shishizun Automation Technology Co., Ltd., Shenzhen, China) with a temporal resolution of 1 ms was used to record the dynamic ablation process and material ejection from the target surface. This system allowed real-time observation of plasma plume evolution and molten droplet ejection during laser ablation. In addition, a high-speed infrared pyrometer (KLEIBER KMGA740-LO, Bremen, Germany) with a temporal resolution of 10 μs and a measurable temperature range of 600–3800 K was employed to monitor the transient temperature evolution of the metal target throughout the entire combined pulse laser ablation process. The high-speed infrared pyrometer was calibrated to measure the average temperature within a circular region of interest (ROI) with a fixed diameter of 1 mm on the target surface, which is consistent with the irradiation spot size of the CW laser. This defined ROI precisely corresponds to the laser–material interaction zone, ensuring that the recorded temperature evolution accurately reflects the thermal state of the active ablation region rather than the surrounding solid metal surface. The temporal evolution of the target temperature reported in this work therefore reflects the thermal state of the molten interaction zone rather than the surrounding solid surface. The temperature represents the average value within the infrared measurement region corresponding to the CW laser spot on the target surface. The high-speed imaging system and the infrared pyrometer were integrated into the experimental optical path, as illustrated in Figure 1a.

Considering the shot-to-shot fluctuation inherent to laser-induced plasma and molten pool dynamics, each ablation condition was repeated five times, and the reported values represent the mean of these measurements. The standard deviation obtained from repeated measurements is now shown as error bars in figure. This allows visualization of the experimental scatter associated with plasma fluctuations and melt dynamics.

## 3. Results and Discussions

### 3.1. Morphological Characteristics of the Metal Target After Laser Ablation

#### 3.1.1. Ablation Morphology Characteristics Under Single CW Laser, Single ns and Combined Pulse Laser Irradiation

**Figure 2:**
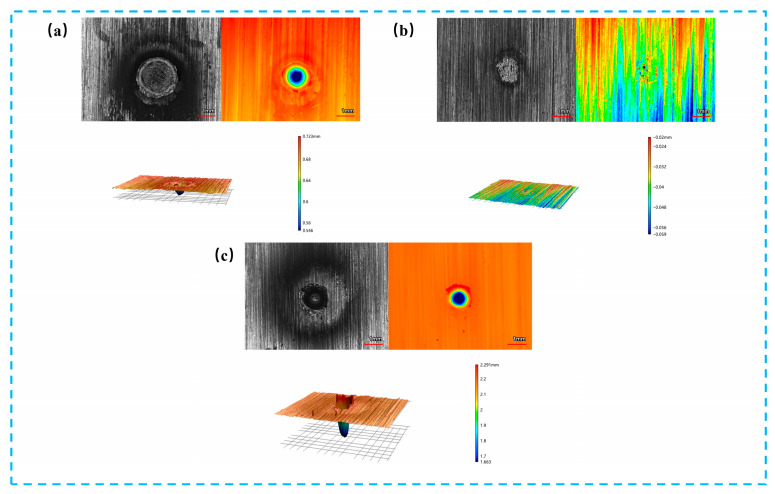
Three-dimensional morphological features after ablation under different laser modes: (**a**) Morphological characteristics of the metal target after ablation by a single 500 W CW laser; (**b**) Morphological characteristics of the metal target after ablation by a single 0.4 J ns laser; (**c**) Morphological characteristics of the metal target after ablation by a combined pulse laser composed of a 500 W CW laser and a 0.4 J nanosecond pulsed laser.

Figure 2 shows the surface morphologies of 304 stainless steel targets after ablation by a single CW laser, a single ns laser and a combined pulse laser, respectively. As shown in Figure 2a, after irradiation with a single 500 W CW laser, the ablated region exhibits a concentric-ring pattern, and the three-dimensional surface morphology reveals a relatively regular ablation crater with no obvious molten material splashing at the crater edge. A slight raised rim is observed at the crater boundary, which is attributed to the outward flow and rapid solidification of molten metal, forming a typical recast layer. These features indicate that material damage under single CW laser irradiation is mainly governed by thermal accumulation and molten metal flow. As shown in Figure 2b, under single 0.4 J nanosecond (ns) laser irradiation, a well-defined circular crater is formed on the target surface. A slight rim uplift is observed along the crater periphery, while no evident lateral melt spreading can be detected. This morphology indicates that the laser energy is predominantly confined to the near-surface region within the pulse duration. The absence of extensive melt pool development suggests that the interaction process is governed by transient melting and rapid solidification, rather than sustained thermal accumulation or prolonged molten layer formation. Additional observations indicate that increasing the nanosecond pulse energy from 0.4 J to 1.0 J does not result in significant changes in the overall ablation morphology. The crater characteristics and dominant ablation features remain generally similar. Therefore, the 0.4 J condition is retained as the representative nanosecond irradiation case for comparison with the CW and combined pulse laser irradiation results. In contrast, as shown in Figure 2c, combined pulse laser ablation results in a markedly enlarged ablated area. The ablation crater exhibits steeper sidewalls and greater depth, accompanied by pronounced molten material splashing and irregular protrusions around the crater edge, indicating the occurrence of spallation behavior. This suggests that the damage mechanism is significantly altered by the introduction of the nanosecond pulsed laser. Notably, the enhanced ablation characteristics observed here imply a strong dependence on the nanosecond pulse energy, which will be further discussed in the following section.

#### 3.1.2. Three-Dimensional Morphological Characteristics of Metal Targets After Combined Pulse Laser Ablation Under Different Nanosecond Pulse Energies

**Figure 3:**
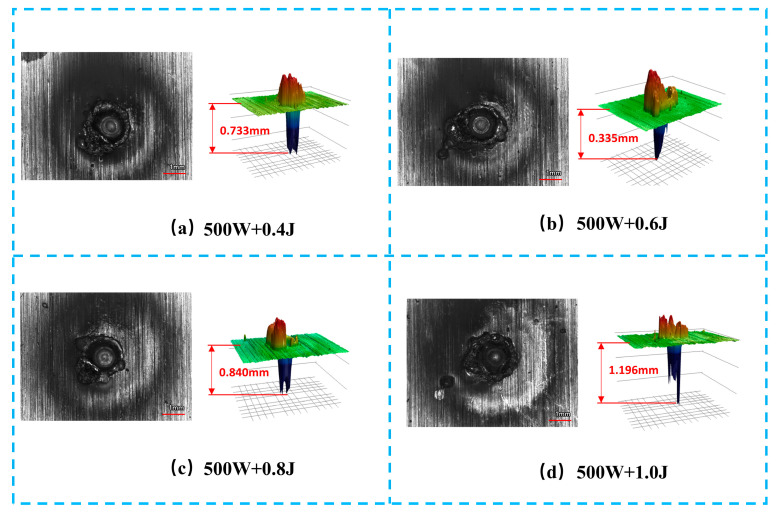
Three-dimensional morphological characteristics of metal targets after combined pulse laser ablation under different nanosecond pulse energies: (**a**) 500 W + 0.4 J; (**b**) 500 W + 0.6 J; (**c**) 500 W + 0.8 J; (**d**) 500 W + 1.0 J.

Figure 3 shows the three-dimensional surface morphologies of metal targets ablated by the combined pulse laser under different nanosecond pulse energies, while the CW laser power is maintained at 500 W. As the nanosecond pulse energy increases from 0.4 J to 1.0 J, corresponding to a fluence of approximately 1.3–3.2 kJ/cm^2^, the ablation morphology exhibits a clear energy-dependent evolution. At a pulse energy of 0.4 J, the ablation crater depth is 0.733 mm, and the raised rim at the crater edge is relatively gentle, resulting in an overall smooth morphology, indicating limited material removal dominated by surface ablation. When the pulse energy increases to 0.6 J, the crater depth decreases to 0.335 mm, whereas the ablated area expands significantly, accompanied by steeper and more irregular edge protrusions, suggesting enhanced molten material splashing and recast layer formation. With further increases in nanosecond pulse energy to 0.8–1.0 J, the ablation crater depth increases again, reaching 1.196 mm, while the height of the edge protrusions decreases and the edge morphology becomes relatively smoother and more regular. This behavior indicates that, at higher pulse energies, a larger fraction of molten material is expelled away from the ablation crater rather than accumulating at the edge. Overall, these results demonstrate that the nanosecond pulse energy plays a crucial role in regulating the three-dimensional ablation morphology and the material damage response during combined pulse laser ablation.

#### 3.1.3. Variation in Ablation Depth of Metals Induced by Combined Pulse Laser Irradiation Under Different Nanosecond Energy Levels

**Figure 4:**
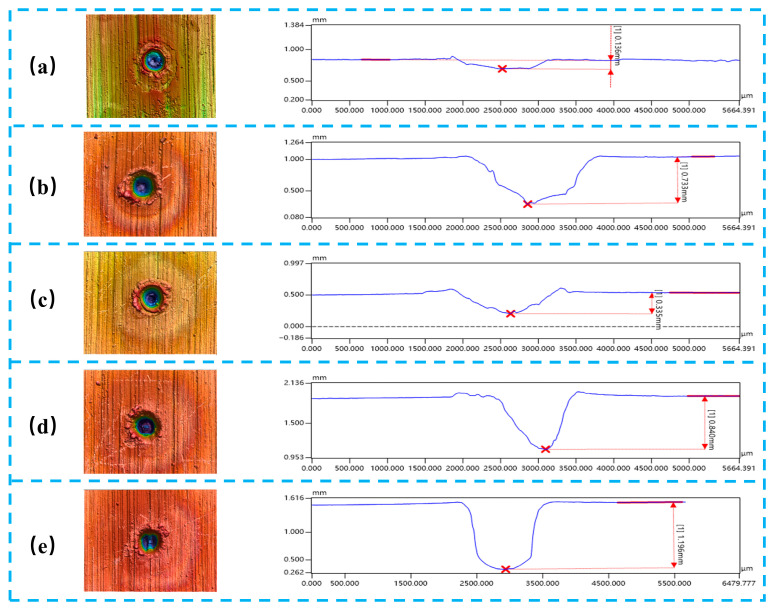
The variation in ablation depth of metals after combined pulse laser irradiation under different nanosecond energy levels: (**a**) 500 W; (**b**) 500 W + 0.4 J; (**c**) 500 W + 0.4 J; (**d**) 500 W + 0.6 J; (**e**) 500 W + 0.6 J. The results shown in figure are transformed into the diagrams in the last figure.

Figure 4 illustrates the variation in ablation depth of metallic targets processed by combined pulse laser irradiation under different nanosecond pulse energy conditions. When the nanosecond pulse energy is 0 J, corresponding to irradiation by a 500 W CW laser alone, the ablation depth is limited to approximately 0.136 mm. Under this condition, only a shallow and well-defined crater is formed on the metal surface, characterized by a relatively smooth morphology and slightly raised edges. When the nanosecond pulse energy increases to 0.4 J, the ablation depth rises to about 0.733 mm, indicating that the introduction of nanosecond pulses induces moderate surface damage while the overall material removal remains limited. As the nanosecond pulse energy further increases to 0.6 J, the ablation depth decreases to approximately 0.335 mm, representing the minimum depth among all combined pulse laser conditions investigated. In this case, molten splashes appear at the crater edges and the recast layer becomes thicker, suggesting that enhanced plasma formation leads to a stronger plasma shielding effect. The formation of this effective “energy barrier” reduces the efficiency of laser energy transfer to the metal target, thereby resulting in a decrease in ablation depth despite the increase in incident laser energy. When the nanosecond pulse energy reaches 0.8–1.0 J, a pronounced increase in ablation depth is observed, with depths of approximately 0.840 mm and 1.196 mm, respectively. Meanwhile, the crater edge roughness increases significantly, and a distinct recast layer together with an evident heat-affected zone develops along the crater walls. This behavior can be attributed to the increase in nanosecond pulse energy, which induces non-uniform expansion of the plasma plume and a reduction in local plasma density. Consequently, the plasma shielding effect is partially alleviated, allowing more laser energy to be absorbed by the plasma and subsequently transferred to the target via inverse bremsstrahlung mechanisms. In addition, the mechanical stress generated by plasma-induced shock waves further promotes material fragmentation and ejection, thereby significantly enhancing the overall ablation efficiency. Overall, with increasing nanosecond pulse energy, the ablation depth exhibits a non-monotonic trend characterized by an initial decrease followed by a substantial increase, demonstrating that nanosecond pulse energy plays a critical role in regulating the ablation behavior during combined pulse laser processing.

### 3.2. Temperature Evolution Curves During Laser Ablation of Metallic Targets

The results presented in Section 3.1 demonstrate that the introduction of nanosecond pulses significantly influences the ablation depth and surface morphology of metallic targets under combined pulse laser irradiation. Since laser–matter interaction and material removal during ablation are inherently governed by thermal processes, it is essential to further examine the temperature evolution of the target during laser irradiation. Therefore, in this section, the transient temperature response of the metallic target under different laser irradiation modes is systematically investigated.

#### 3.2.1. Temperature Evolution Curves During Ablation Under CW Laser Irradiation and Combined Pulse Laser Irradiation

**Figure 5:**
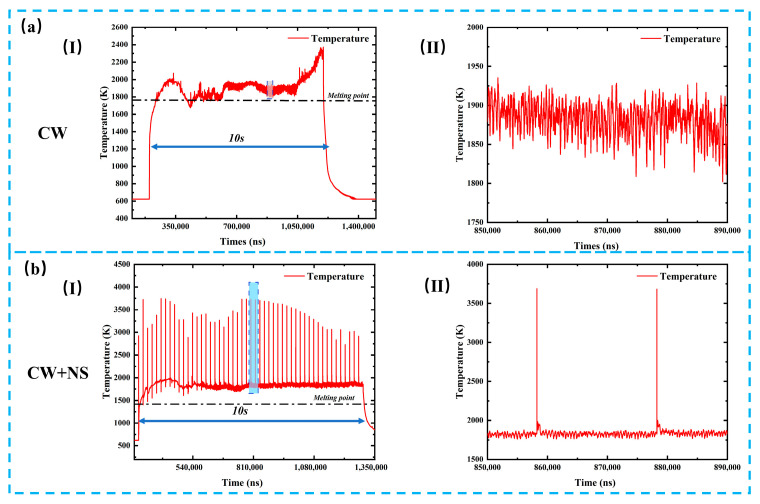
Transient temperature evolution curves of metallic targets during ablation under CW laser irradiation and combined pulse laser irradiation: (**a-I**) the temperature evolution curve during ablation under a 500 W CW laser alone; (**a-II**) enlarged view of the blue region in the temperature evolution curve during ablation under a 500 W CW laser alone; (**b-I**) the temperature evolution curve during ablation under combined pulse laser irradiation; (**b-II**) enlarged view of the blue region in the temperature evolution curve during ablation under combined pulse laser irradiation.

Figure 5 presents the transient temperature evolution of metallic targets during ablation under CW laser irradiation and combined pulse laser irradiation. As shown in Figure 5(a-I), under irradiation by a 500 W CW laser alone, the target temperature increases gradually with irradiation time, and the peak temperature remains below 2600 K. After a period of heating, temperature fluctuations within the range of approximately 1800–1950 K are observed, as illustrated in Figure 5(a-II). This behavior indicates that, under CW laser irradiation, the metal target continuously absorbs thermal energy, leading to temperature rise and the formation of molten metal, which flows within the ablation region and exposes a fresh metal surface. Although heat is dissipated during this process, the sustained laser irradiation enables the newly exposed metal to absorb energy again, resulting in repeated heating–cooling cycles and thus temperature oscillations. Meanwhile, the molten metal flows toward the crater edges and resolidifies to form a recast layer. As the CW laser continues to deliver energy while heat dissipation remains limited, the temperature increases continuously during the later stage of irradiation until the laser is switched off, after which the temperature rapidly decreases. In contrast, under combined pulse laser irradiation, the temperature evolution exhibits significantly different characteristics, as shown in Figure 5(b-I). Upon the incidence of nanosecond laser pulses, the target temperature rises abruptly, reaching peak values exceeding 3500 K, which are substantially higher than those observed under CW laser ablation. After reaching the peak, the temperature decreases rapidly and subsequently exhibits small-amplitude fluctuations, as shown in Figure 5(b-II). Under the combined action of CW and nanosecond lasers, the nanosecond pulses effectively remove the continuously heated molten metal through impulsive interaction, accompanied by the ejection of molten droplets. This results in transient temperature rises and drops synchronized with the nanosecond pulse irradiation. After the molten material is removed, a new and relatively cooler metal layer is exposed, which then absorbs energy from the CW laser, leading to remelting and flow of molten metal toward the crater edges. Consequently, small temperature fluctuations are observed between successive nanosecond laser pulses.

#### 3.2.2. Temperature Evolution Curves During Combined Pulse Laser Ablation Under Different Nanosecond Pulse Energy Levels

**Figure 6:**
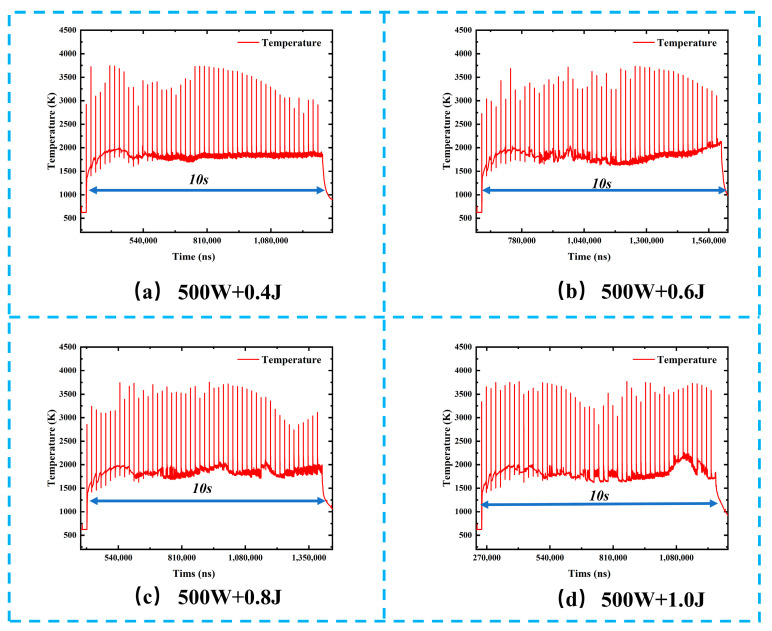
The temperature evolution curves during combined pulse laser ablation under different nanosecond pulse energy levels: (**a**) 500 W + 0.4 J; (**b**) 500 W + 0.6 J; (**c**) 500 W + 0.8 J; (**d**) 500 W + 1.0 J.

Figure 6 shows the transient temperature evolution of metallic targets during combined pulse laser ablation under different nanosecond pulse energy conditions. At the initial stage of combined pulse laser irradiation, the target surface rapidly heats up due to the absorption of CW laser energy, and the temperature reaches a relatively stable plateau at approximately 400 μs. This stage is mainly dominated by the thermal effect of the CW laser. During the stable plateau stage of combined pulse laser irradiation, within the 10 s laser exposure duration, the temperature remains at a baseline platform of approximately 1500–2000 K, superimposed with high-frequency and intense fluctuations induced by the nanosecond laser pulses, with peak temperatures exceeding 3500 K. The coexistence of a stable temperature platform and superimposed pulse-induced fluctuations reflect the characteristic ablation mechanism of combined pulse laser processing, in which CW laser irradiation provides sustained thermal input, while nanosecond pulses deliver instantaneous energy enhancement. As the nanosecond pulse energy increases from 0.4 J to 1.0 J, the average temperature of the stable plateau gradually rises from approximately 1720 K to about 1900 K. This trend indicates that higher nanosecond pulse energy promotes greater thermal accumulation in the ablation region, thereby reducing the thermal response threshold of the material and creating a more favorable thermal environment for subsequent pulse-enhanced ablation. In addition, the peak temperature associated with pulse-induced fluctuations increases with nanosecond pulse energy. At a pulse energy of 0.4 J, the temperature fluctuation peaks reach approximately 3500 K, whereas at 1.0 J, the peak temperature approaches 3700 K. This observation directly reflects the positive correlation between nanosecond pulse energy and instantaneous thermal shock intensity. Higher pulse energy enables the material surface to reach higher temperatures within an extremely short time, thereby intensifying surface melting, vaporization, and even plasma ejection phenomena.

Overall, the combined pulse laser ablation mechanism relies on CW laser irradiation to maintain the target surface at an elevated temperature platform through sustained preheating, effectively lowering the melting and vaporization thresholds of the material. Superimposed nanosecond pulses then provide high-energy, short-duration thermal shocks on the basis of accumulated heat, further aggravating thermal damage. Increasing the nanosecond pulse energy leads to stronger instantaneous thermal effects within the same irradiation duration, manifested by higher temperature peaks and larger fluctuation amplitudes. These results demonstrate that precise regulation of nanosecond pulse energy enables effective control of the thermal behavior in the ablation zone, thereby offering a viable approach for optimizing ablation efficiency and processing quality.

In our experiments, the CW laser preheating increased the surface temperature above the melting point of 304 steel (T_m_ = 1450 K), as confirmed by infrared thermography. In addition, high-speed imaging shows a stable molten pool exhibiting liquid flow and surface fluctuation before the arrival of the nanosecond pulses. Prior to nanosecond irradiation, CW laser heating produced a stable molten pool with surface temperature exceeding the melting point of steel, indicating that the ns pulses interacted with a fully molten target surface. These observations indicate that the ns laser interacts with a fully molten surface rather than a near-molten state.

### 3.3. High-Speed Imaging of the Metal Target During the Ablation Process

The results presented in Section 3.2 highlighted the significant thermal effects observed during combined pulse laser ablation, particularly with regard to temperature evolution and its influence on material removal mechanisms. To further understand the dynamics of the ablation process, high-speed imaging was employed to visualize the plasma plume and molten material behavior during ablation. Section 3.3 delves into the high-speed imaging analysis of metal targets subjected to CW laser irradiation, providing detailed insights into the temporal evolution of the plasma plume and the underlying physical phenomena during laser-material interactions.

#### 3.3.1. High-Speed Imaging of Metal Target Ablation Under CW Laser Irradiation

**Figure 7:**
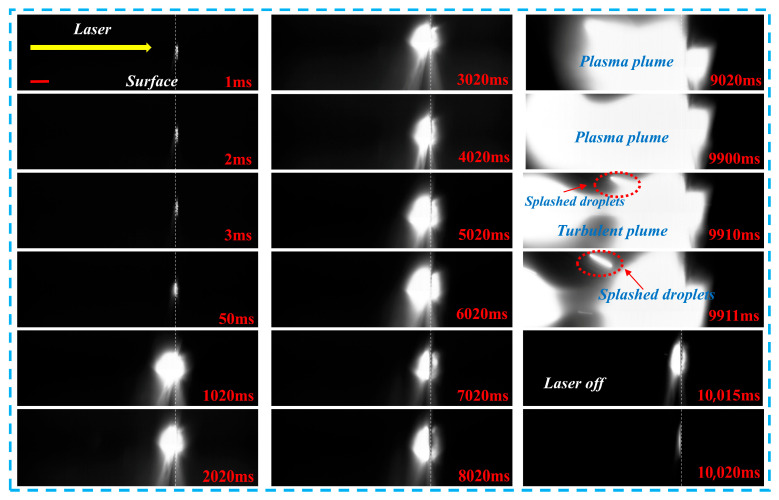
High-speed imaging of metal target ablation under CW laser irradiation, with a scale bar of 1 mm.

Figure 7 presents high-speed imaging of metal target ablation under CW laser irradiation, with a scale bar of 1 mm. During the initial ablation stage (0–49 ms), the laser spot begins to interact with the target surface. As the temperature rises and surface melting initiates, a weak luminous region forms. At 50 ms, the brightness on the target surface increases significantly, indicating an accelerated material vaporization rate and the formation of the initial plasma plume. The plume remains tightly attached to the target surface, exhibiting a constrained ejection state. From 1020 to 2020 ms, the plasma plume displays a bright, collimated morphology and ejects downward at high speed, which marks the stable plasma ejection stage under CW heating. At this point, the plume dynamics are primarily governed by heat conduction and directional movement. Between 3020 and 8020 ms, the brightness of the plume remains high, while its radial dimension gradually expands. This expansion indicates that the plasma is interacting and diffusing with the surrounding ambient gas, causing the plume morphology to transition from a collimated shape to a hemispherical form, showing clear signs of thermal expansion. At 9020 ms, the top of the plasma plume exhibits an irregular bifurcation structure, signifying the transition to a turbulent evolution stage. Between 9900 and 9911 ms, the plume shows clear turbulent features due to intensified interactions between internal temperature gradients, velocity gradients, and surrounding gas. These interactions lead to flow instability and the formation of complex turbulent structures. As a result, the luminous region of the plume expands and becomes more diffuse, and distinct trajectories of splashed droplets are observed. After 10,000 ms, when the laser is switched off, the plasma plume loses its continuous energy supply. The brightness decays rapidly, leaving only weak residual luminescence, signaling rapid plasma cooling and recombination, followed by the rapid dissipation of the plume structure. At 10,015 ms, a bright layer appears on the target surface, corresponding to the recast layer formed by the flow and re-solidification of molten metal. Throughout the entire process, no significant droplet splashing is observed, suggesting that ablation under CW laser irradiation is primarily governed by heat accumulation. The observed splashing of droplets is attributed to the shock pressure generated by the plasma plume acting on the molten region. The plasma plume remains bright between 1020 and 8020 ms, exhibiting intermittent brightness fluctuations. This behavior correlates with the observed temperature fluctuations in the temperature evolution curves. The appearance of plasma turbulence between 9020 and 9911 ms indicates that the metal target is in a combustion-like state. This stage necessitates continuous heat absorption, explaining the sustained increase in temperature observed in the later stages of the temperature evolution curve.

#### 3.3.2. High-Speed Imaging of Metal Target Ablation Under Combined Pulse Laser Irradiation

**Figure 8:**
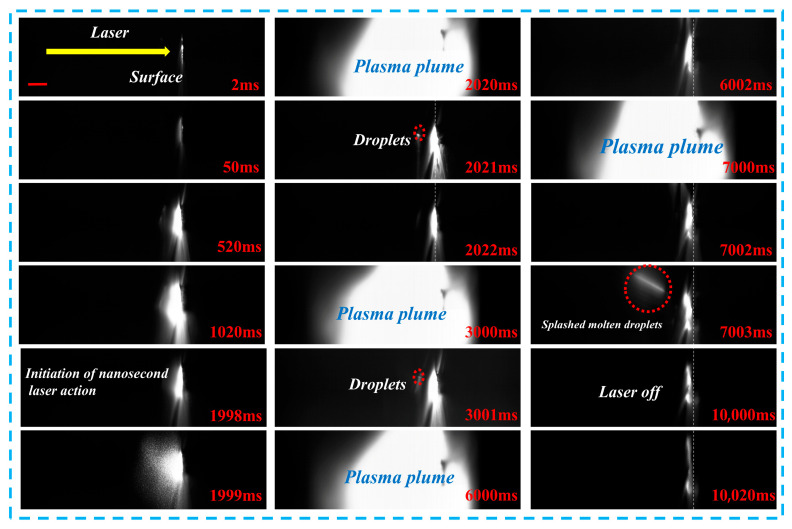
High-speed imaging of metal target ablation under combined pulse laser irradiation, with a scale bar of 1 mm. The nanosecond laser pulses were delivered continuously at 5 Hz (200 ms interval). The time stamps indicate representative frames selected from the high-speed sequence rather than individual pulse events.

Figure 8 presents high-speed imaging of metal target ablation under combined pulse laser irradiation. All results in this section were obtained with a nanosecond pulse energy of 0.4 J. In the initial stage of combined pulse laser ablation, the target surface absorbs the laser energy, which results in a steady increase in temperature and a faint glow emitted from the surface. As the laser irradiation continues, the surface temperature rises above the threshold, initiating rapid material vaporization and the formation of a high-temperature, high-pressure plasma plume that expands outward from the target. Between 1020 and 1999 ms, the plasma plume continues to expand, indicating that the combined pulse laser delivers a uniform energy deposition on the target surface, promoting plasma formation and expansion. At 2020–2022 ms and 3000–3001 ms, periodic oscillations are observed in the plasma plume, characterized by rapid expansion followed by brief contraction and another expansion. These fluctuations are synchronized with the instantaneous effects of the nanosecond pulse laser, corresponding to the sharp rises and falls in the temperature curve. During the contraction phases at 2021 ms and 3001 ms, small molten droplets are ejected from the target surface. These splashed droplets are caused by the abrupt pressure changes in the plasma, which act upon the molten material, confirming the significant migration of liquid-phase material during the ablation process. Between 7000 and 7003 ms, the plasma plume reappears, and at 7003 ms, larger splashed molten droplets are observed. This suggests that the later pulses of the combined pulse laser disturb the molten pool formed in the earlier stage, resulting in intensified material ejection. Following the cessation of laser irradiation at 10,000 ms, the plasma plume dissipates rapidly within 1–2 ms, and the brightness from the target surface diminishes quickly. At 10,020 ms, only weak residual thermal radiation remains, indicating that the target enters a rapid cooling phase, followed by the solidification of the molten pool and the formation of a recast layer. Throughout the ablation process, intense droplet splashing is observed, which indicates that the damage induced by combined pulse laser ablation is primarily driven by the instantaneous pressure shock from the plasma. This observation further demonstrates that combined pulse laser significantly enhances the ability to cause surface damage and material removal from metal targets.

#### 3.3.3. High-Speed Imaging of Metal Target Ablation Under Combined Pulse Laser Irradiation at the Same Time Points but with Different Nanosecond Pulse Energies

**Figure 9:**
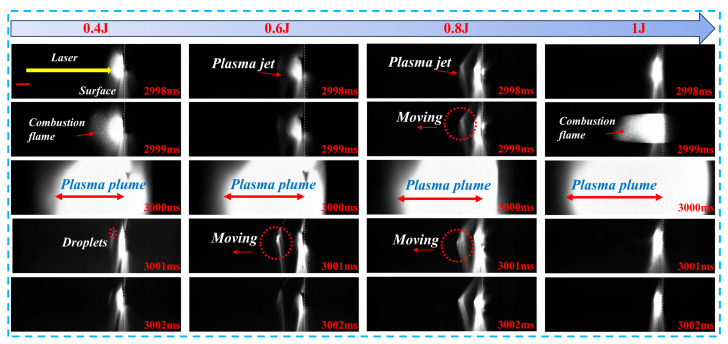
High-speed imaging of metal target ablation under combined pulse laser irradiation at the same time points but with different nanosecond pulse energies, with a scale bar of 1 mm.

Figure 9 presents high-speed imaging that captures the evolution of key physical phenomena during combined pulse laser ablation of metal targets, including the formation of plasma jets, combustion flame propagation, plasma plume expansion, and droplet ejection. At 2998 ms, the target surface begins to emit significant brightness as it absorbs laser energy and heats up. With increasing nanosecond pulse energy, a plasma jet forms, and both the brightness and size of the jet increase significantly with higher energy levels. At 2999 ms, under the 0.4 J condition, a combustion flame begins to spread, exhibiting typical oxidation combustion characteristics. At 0.6 J, the plasma jet remains dominant, with weaker flame signals observed. At 0.8 J, the plasma jet begins to exhibit lateral movement, and flame formation starts at the edges, indicating enhanced interaction between the plasma and the surrounding gas. At 1.0 J, a large, continuous combustion flame forms, covering the downstream region of the plasma jet, indicating more efficient energy deposition by the high-energy plasma, which promotes the mixing combustion of evaporated products with surrounding gases. At 3000 ms, large plasma plumes are observed under all nanosecond pulse energy conditions. The lateral size of the plasma plume increases with higher energy, suggesting that greater surface energy leads to stronger plasma expansion and, consequently, more intense damage to the metal target surface. In the later stages (3001–3002 ms), droplet ejection is observed under the 0.4 J condition. At 0.6 J and 0.8 J, the plasma jet continues to exhibit lateral movement, while at 1.0 J, the target surface remains brightly lit, and the combustion flame persists, indicating that the post-ablation phase under high energy is dominated by combustion reactions. Overall, with increasing nanosecond pulse energy, the expansion capability of the plasma plume is significantly enhanced, leading to more droplet ejection and plasma jet formation. This demonstrates that effective control of nanosecond pulse energy can significantly improve the ablation efficiency of metal targets.

### 3.4. Comparison of Ablation Performance Under Different Condition Parameters

Section 3.3 provides a detailed analysis of the high-speed imaging results, which illustrate the evolution of key physical phenomena such as plasma jet formation, combustion flame propagation, and droplet ejection during combined pulse laser ablation. To further understand the underlying mechanisms behind these observed phenomena, Section 3.4 shifts focus to a comparative analysis of the ablation performance under varying energy conditions. This section will assess how different laser parameters influence the ablation depth, material removal rate, and overall efficiency of the process.

**Figure 10:**
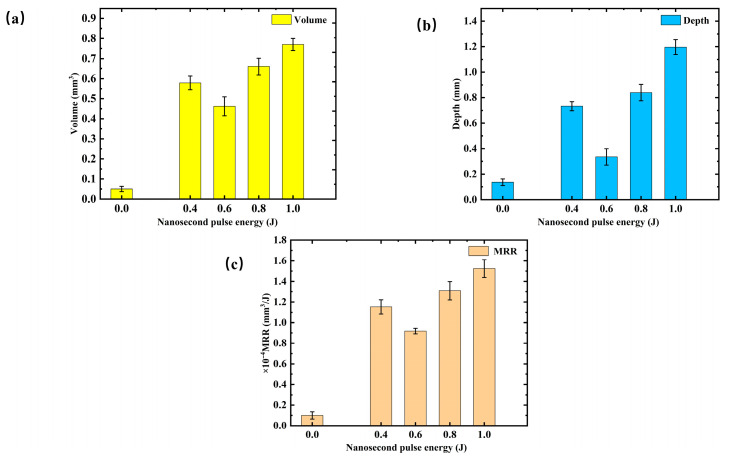
Comparison of laser ablation performance under different condition parameters: (**a**) volume comparison; (**b**) depth comparison; (**c**) material removal rate (MRR) comparison.

Figure 10 compares the laser ablation performance under different condition parameters: (a) volume, (b) depth, and (c) material removal rate (MRR). Regarding volume removal, when the pulse energy is 0.0 J, the ablation volume is nearly 0, only about 0.05 mm^3^. As the energy increases from 0.2 J to 0.4 J, the ablation volume rises sharply, reaching a peak of approximately 0.579 mm^3^ at 0.4 J. Further increases in energy cause the ablation volume to first decrease and then increase, with a final value of around 0.770 mm^3^ at 1.0 J. This behavior indicates that, in the low energy range, increasing energy significantly enhances ablation. However, beyond a certain threshold, plasma shielding effects and thermal saturation limit further increases in ablation volume. The ablation depth consistently increases with higher pulse energy. From 0.0 J to 1.0 J, the depth grows from about 0.136 mm to 1.196 mm, exhibiting a trend of initial decrease followed by an increase. The changes in material removal rate (MRR) align with the ablation volume. At 0.0 J, the MRR is only 0.1 × 10^−4^ mm^3^/J, and it increases significantly to 1.153 × 10^−4^ mm^3^/J at 0.4 J. As energy continues to rise, the MRR initially decreases before increasing again, reaching 1.524 × 10^−4^ mm^3^/J at 1.0 J. When using only 500 W CW irradiation, the removal volume is 0.05 mm^3^, with an average depth of 0.136 mm and an MRR of 0.1 × 10^−4^ mm^3^/J. In contrast, with the introduction of nanosecond pulses, the average removal volume increases to 0.618 mm^3^, the depth to 0.776 mm, and the MRR to 1.226 × 10^−4^ mm^3^/J. Overall, the addition of nanosecond pulses significantly enhances the ablation performance, with a clear trend of first decreasing and then increasing values, as compared to CW laser ablation.

Experiments with intentional lateral offset between the CW spot and the nanosecond filament were performed to directly evaluate the effect of focus mismatch, even a small offset produces clearly asymmetric and laterally displaced craters that differ markedly from the symmetric morphology observed in the main dataset. This demonstrates that any significant focus misalignment would be readily identifiable in the ablation patterns. Since the craters in these study figures remain symmetric and centered for all pulse energies, the observed efficiency trends cannot be attributed to focus fluctuation.

In the present regime, however, the nanosecond pulse propagates in air under filamentation with power-clamping behavior. As pulse energy increases, the filament diameter and interaction length increase while the peak intensity remains nearly constant. Consequently, the effective interaction diameter on the molten pool varies with pulse energy, leading to different shock loading and melt expulsion conditions. The ablation efficiency therefore reflects a competition between plasma-mediated shielding along the filament channel and hydrodynamic melt removal governed by the interaction area, rather than focusing instability.

### 3.5. Comprehensive Discussion

The combined pulse laser ablation of metal targets, using a combination of CW laser and nanosecond pulses, achieves a synergistic effect that combines the preheating capability of the CW laser and the instantaneous ablation enhancement from the nanosecond pulses. This synergy is driven by the temporal and energy complementarity between the two lasers, altering the thermal state of the target material and the evolution of the plasma, ultimately enhancing the ablation efficiency.

During the initial stage of combined pulse laser ablation, the metal target absorbs energy from the CW laser, causing a gradual rise in temperature. The heat diffuses into the interior through thermal conduction, bringing the surface of the target to a near-molten state. This process lowers the ablation threshold, creating a thermal accumulation layer on the target surface. As observed in high-speed imaging, this leads to a significant glow on the target surface in the early stages of laser irradiation. Following this thermal accumulation, the nanosecond pulse laser triggers a rapid temperature rise, causing the ablation zone to undergo rapid melting, vaporization, and plasma jet formation. The shockwave generated during this process directly impacts the molten region, causing material to be ejected in the form of droplets. These splashed droplets are a result of the competition between the plasma jet’s shockwave pressure and the surface tension of the molten material. As plasma pressure increases, the molten material is “blown off” the target surface, forming droplets. During the intervals between nanosecond pulses, the pressure drops sharply, and surface tension pulls some droplets back toward the target’s molten region. This cyclical process is observed in high-speed imaging, with droplets appearing and disappearing as the plasma plume fluctuates.

The CW continuously supplies thermal energy to the molten pool, maintaining the temperature between the melting and boiling points. Each nanosecond pulse triggers an impact that removes molten material, while the CW immediately reintroduces heat, converting the surrounding thermal accumulation layer into a new molten region. This results in a continuous and uniform material removal process. After the laser irradiation ends, both beams shut off simultaneously, and the remaining molten region cools slowly under non-impact conditions, forming a recast layer.

In the combined action of the CW and nanosecond pulse laser, the energy deposition on the target surface varies periodically, leading to periodic fluctuations in the plasma plume. Upon the first nanosecond pulse, the metal target’s molten region rapidly vaporizes, creating a plasma plume. During the intervals between pulses, the plasma plume expands and cools through radiation, causing a decrease in density and weakening of the plume. Subsequently, with each new nanosecond pulse, the weakened plasma plume absorbs energy and expands again, resulting in periodic oscillations in the plasma plume throughout the combined pulse laser ablation process.

This study reveals the evolution of the plasma shielding effect and its regulatory role in nanosecond/continuous-wave (ns/CW) composite laser ablation of metals through the integration of high-speed in situ imaging, infrared transient thermography, and quantitative three-dimensional profilometry. The coupled analysis of plasma plume dynamics, target thermal response, and ablation morphology establishes a multidimensional correlation between plasma behavior and macroscopic ablation performance. Quantitative measurements of ablation volume, depth, and material removal rate under varying nanosecond pulse energies provide direct evidence for a three-stage evolution of plasma shielding (weak–strong–mitigated). The results clarify the intrinsic origin of the non-monotonic ablation performance and demonstrate that plasma shielding governs laser energy coupling and material removal during composite laser ablation.

## 4. Conclusions

This study investigates the plasma shielding effect during the ablation of metal targets under the combined action of CW and nanosecond pulse lasers. An experimental system was developed with a CW laser power of 500 W and nanosecond pulse energies of 0.4 J, 0.6 J, 0.8 J, and 1.0 J. Using high-speed imaging, infrared temperature measurements, and 3D surface morphology characterization, the effects of varying nanosecond pulse energies on the 3D morphology, temperature variations, and dynamic processes of metal target ablation were examined. The main conclusions are as follows:

(1) Single CW ablation: The ablation process is primarily dominated by thermal accumulation, resulting in the formation of regular craters on the target surface, with the edges being raised due to the solidification of molten material.
(2) Synergistic effect of CW and nanosecond Pulses: The combined pulse laser ablation exhibits a significant synergistic effect, combining CW preheating and nanosecond pulse enhancement. CW irradiation raises the target surface temperature to near the melting threshold, while the nanosecond pulses rapidly increase the temperature to above 3500 K, triggering intense melting, vaporization, and plasma formation. Compared to single CW ablation, the combined pulse laser significantly enhances ablation performance, with the maximum ablation volume increasing from 0.05 mm^3^ to 0.770 mm^3^ and the maximum depth increasing from 0.136 mm to 1.196 mm.
(3) Evolution of plasma shielding effect: The plasma shielding effect evolves in three stages: weak shielding, strong shielding, and shielding alleviation, depending on the nanosecond pulse energy. In the weak shielding stage (0.4 J), the plasma plume has low density and small volume, resulting in minimal shielding, allowing efficient laser energy transfer to the target. In the strong shielding phase (0.6 J), high-density plasma forms a shielding layer that absorbs and reflects laser energy, thereby reducing the ablation performance. In the shielding alleviation stage (0.8–1.0 J), the plasma expands outward in a jet-like form, lowering the local density and alleviating the shielding effect, leading to a recovery in ablation performance.
(4) Control of ablation performance via plasma shielding effect: The plasma shielding effect plays a key role in regulating the ablation process. By accurately controlling the nanosecond pulse energy according to the evolution of the plasma shielding effect, the ablation performance of combined pulse lasers can be significantly improved. This approach not only suppresses the negative effects of plasma shielding but also maximizes the synergistic advantages of combined pulse lasers in material ablation.

## Data Availability

The original contributions presented in the study are included in the article; further inquiries can be directed to the corresponding author.
